# Supplementary material for: Overexpression of PtoCYCD3;3 Promotes Growth and Causes Leaf Wrinkle and Branch Appearance in Populus
Source: Int J Mol Sci. 2021 Jan 28;22(3):1288. doi: 10.3390/ijms22031288 (PMC7866192; doi:10.3390/ijms22031288)
Supplement: Supplementary file 1 [file ijms-22-01288-s001.zip › Supplementary Figure S1.pdf]

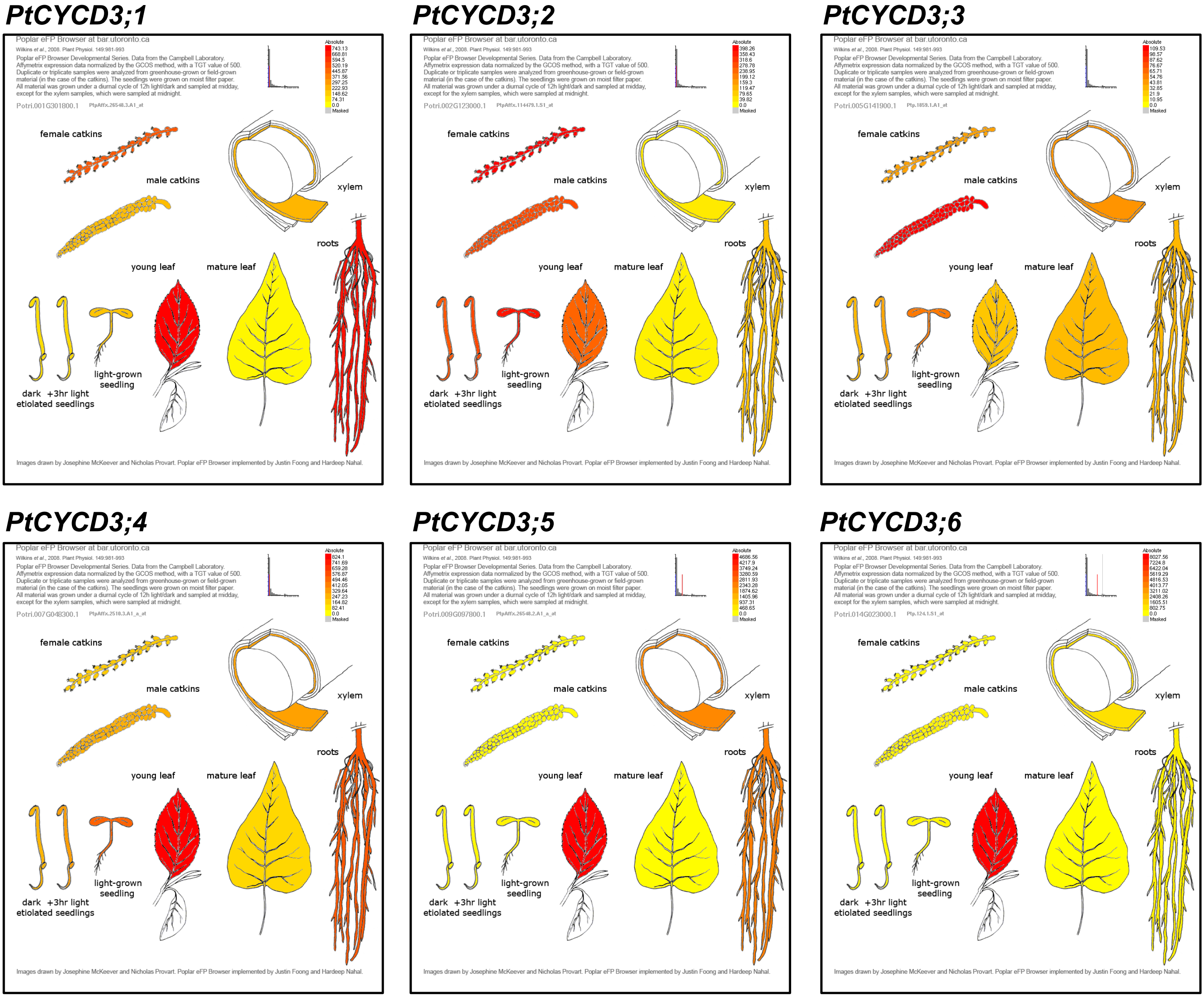

Supplementary Figure S1 Expression of *PtCYCD3* type genes based on Poplar eFP Browser (http://bar.utoronto.ca/efppop/cgi-bin/efpWeb.cgi).
