# Supplementary material for: Overexpression of PtoCYCD3;3 Promotes Growth and Causes Leaf Wrinkle and Branch Appearance in Populus
Source: Int J Mol Sci. 2021 Jan 28;22(3):1288. doi: 10.3390/ijms22031288 (PMC7866192; doi:10.3390/ijms22031288)
Supplement: Supplementary file 1 [file ijms-22-01288-s001.zip › Supplementary Figure S2.pdf]

A (Entire wood-forming region Log_2 expression ratios)

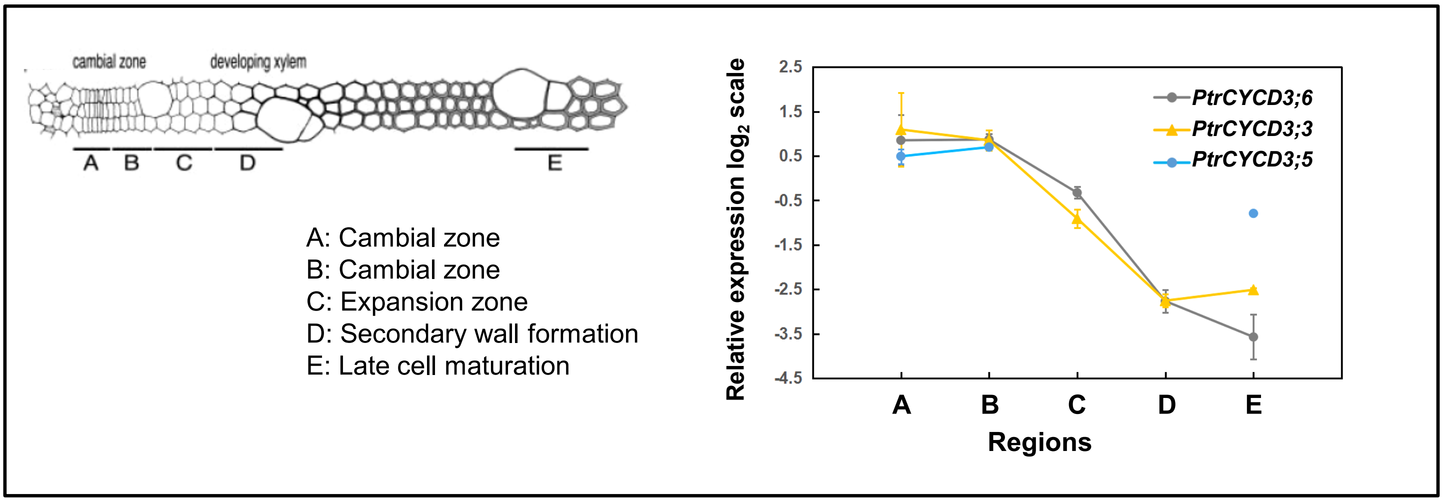

B (CSA/CSB Log_2 expression ratios)

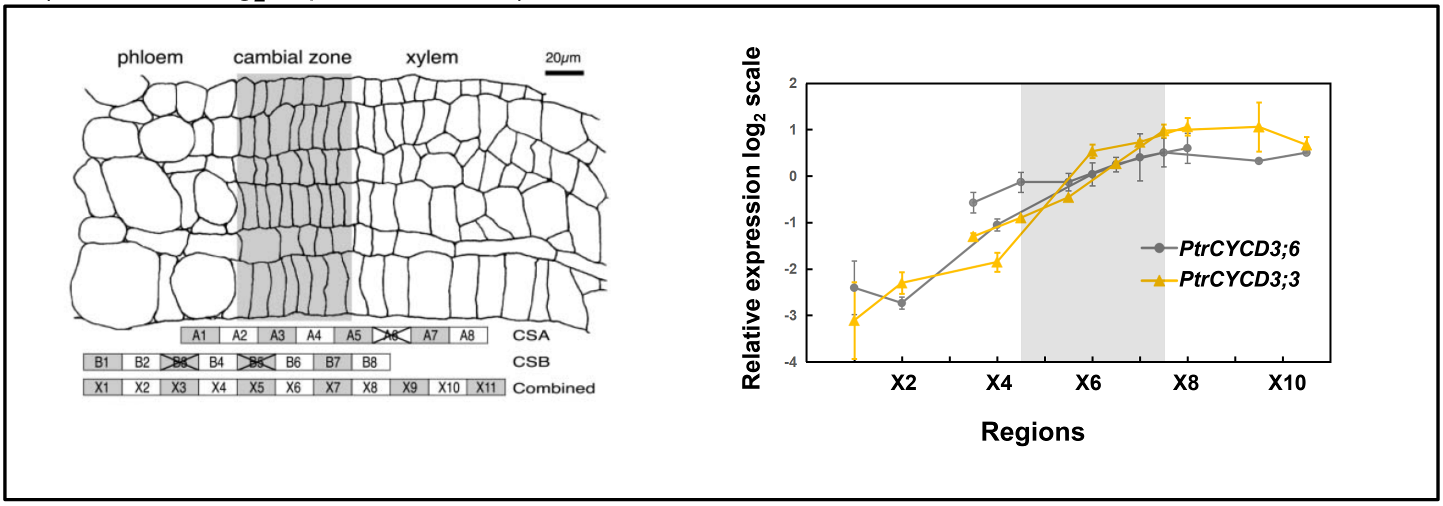

C (Meristem Comparison relative expression)

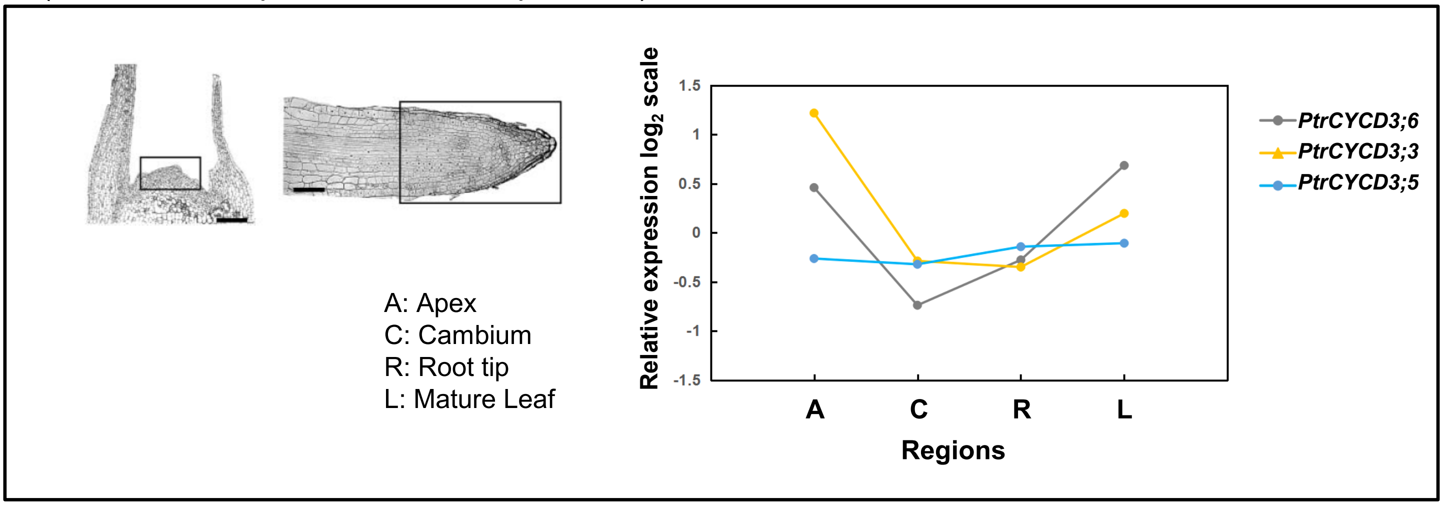

Supplementary Figure S2 Expression of *PtCYCD3* type genes across the wood-forming meristem secitions of *Populus tremula* based on normalized microarray data for Schrader *et al.*,2004.
