# Supplementary material for: Overexpression of PtoCYCD3;3 Promotes Growth and Causes Leaf Wrinkle and Branch Appearance in Populus
Source: Int J Mol Sci. 2021 Jan 28;22(3):1288. doi: 10.3390/ijms22031288 (PMC7866192; doi:10.3390/ijms22031288)
Supplement: Supplementary file 1 [file ijms-22-01288-s001.zip › Supplementary Figure S5.pdf]

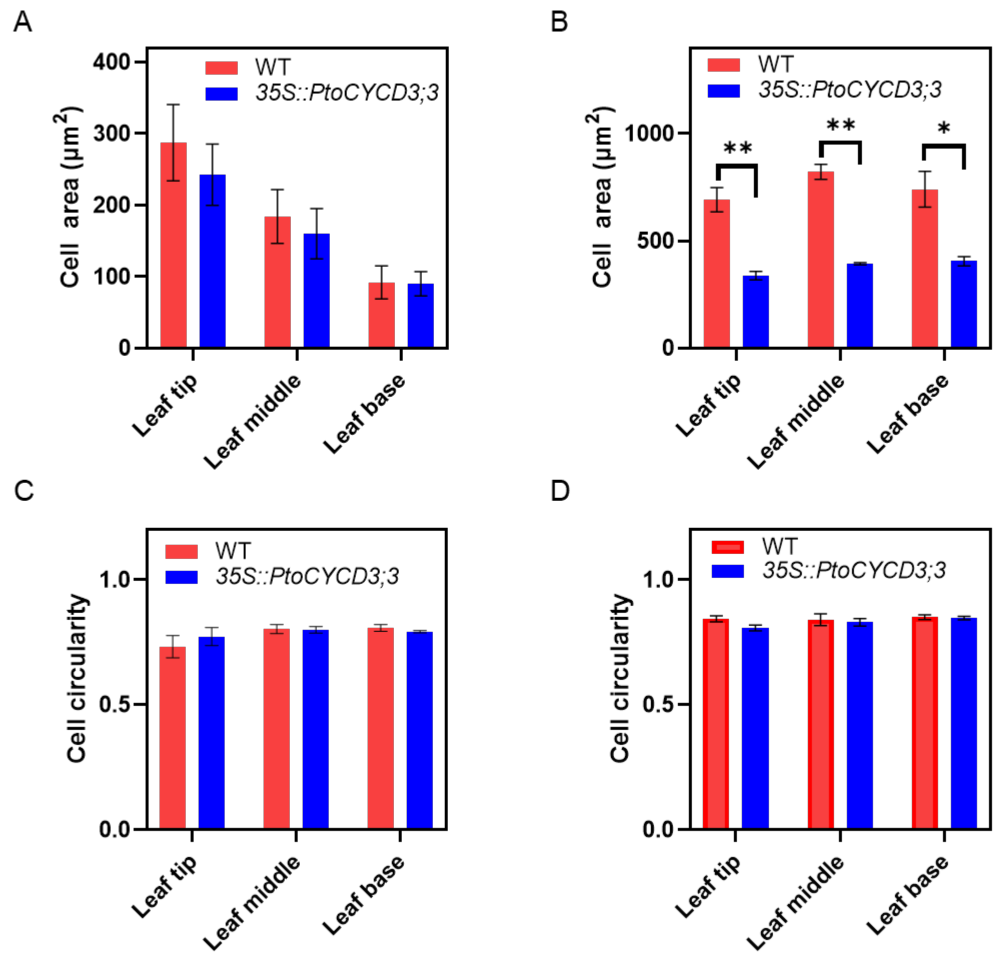

Supplementary Figure S5. Cell area and cell circularity on the adaxial epidermis of wild-type and *35S::PtoCYCD3;3* leaves. (A) Cell area of adaxial epidermis of young leaves; (B) Cell area of adaxial epidermis of mature leaves; (C) Cell circularity of adaxial epidermis of young leaves; (D) Cell circularity of adaxial epidermis of mature leaves.
