# Supplementary material for: Overexpression of PtoCYCD3;3 Promotes Growth and Causes Leaf Wrinkle and Branch Appearance in Populus
Source: Int J Mol Sci. 2021 Jan 28;22(3):1288. doi: 10.3390/ijms22031288 (PMC7866192; doi:10.3390/ijms22031288)
Supplement: Supplementary file 1 [file ijms-22-01288-s001.zip › Supplementary Figure S6.pdf]

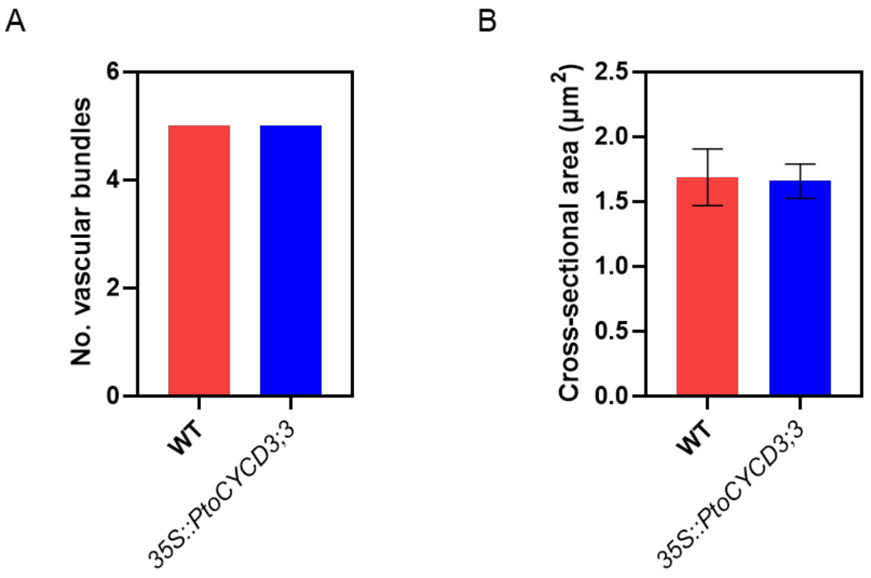

Supplementary Figure S6. Effect of overexpression of *PtoCYCD3;3* on young stems of *Populus*. (A) Number of vascular bundles of the second internode of wild-type and *35S::PtoCYCD3;3* stems. (B) Cross-sectional area of the second internode of wild-type and *35S::PtoCYCD3;3* stems.
