# Supplementary material for: Overexpression of PtoCYCD3;3 Promotes Growth and Causes Leaf Wrinkle and Branch Appearance in Populus
Source: Int J Mol Sci. 2021 Jan 28;22(3):1288. doi: 10.3390/ijms22031288 (PMC7866192; doi:10.3390/ijms22031288)
Supplement: Supplementary file 1 [file ijms-22-01288-s001.zip › Supplementary Figure S7.pdf]

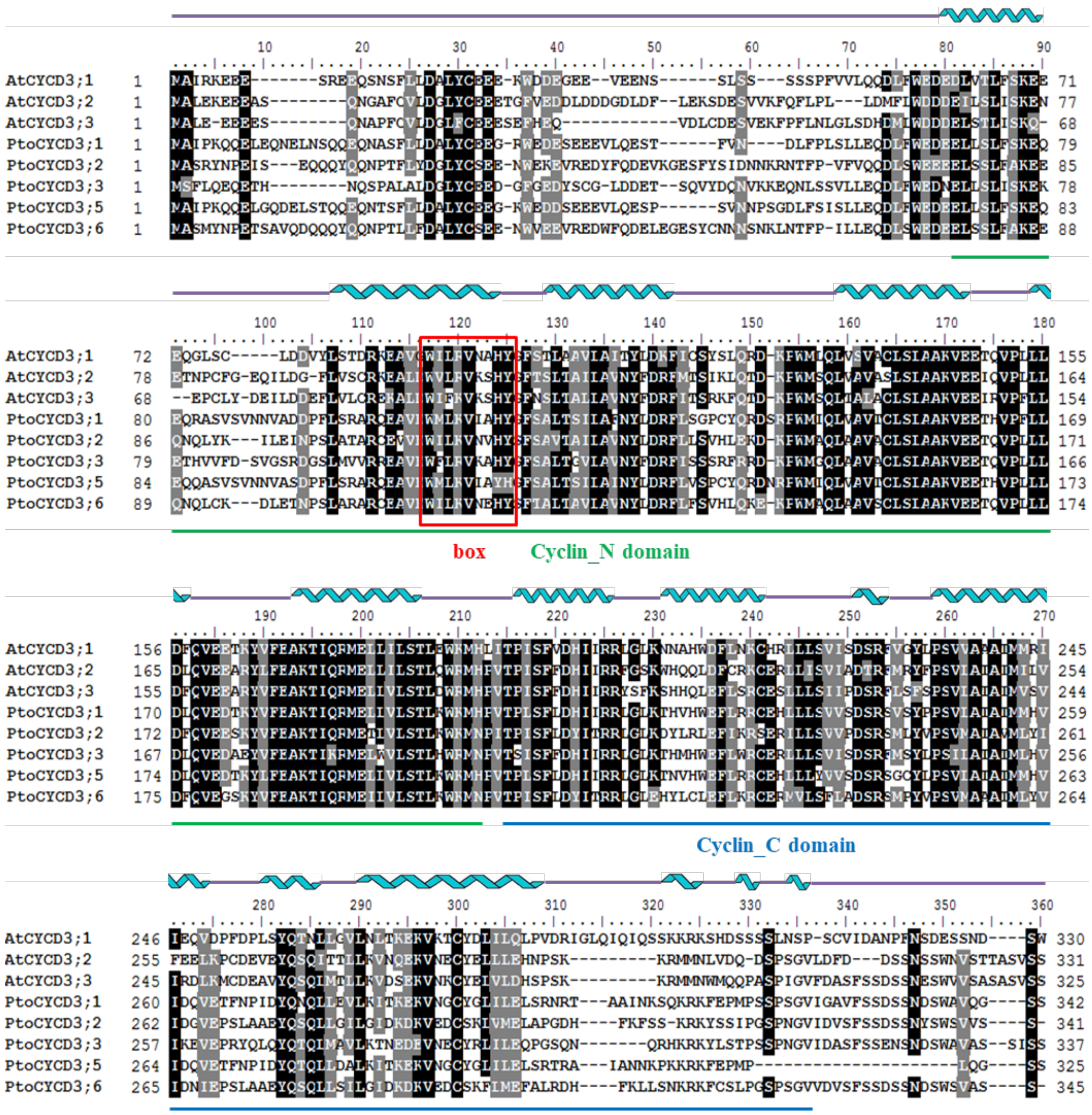

Supplementary Figure S7. *AtCYCD3* and *PtoCYCD3* sequence alignment. The red box indicated the cyclin box of *CYCD* genes; the green line indicated Cyclin_N domain; the blue line indicated Cyclin_C domain.
