# Supplementary material for: Overexpression of PtoCYCD3;3 Promotes Growth and Causes Leaf Wrinkle and Branch Appearance in Populus
Source: Int J Mol Sci. 2021 Jan 28;22(3):1288. doi: 10.3390/ijms22031288 (PMC7866192; doi:10.3390/ijms22031288)
Supplement: Supplementary file 1 [file ijms-22-01288-s001.zip › Supplementary Figure S8.pdf]

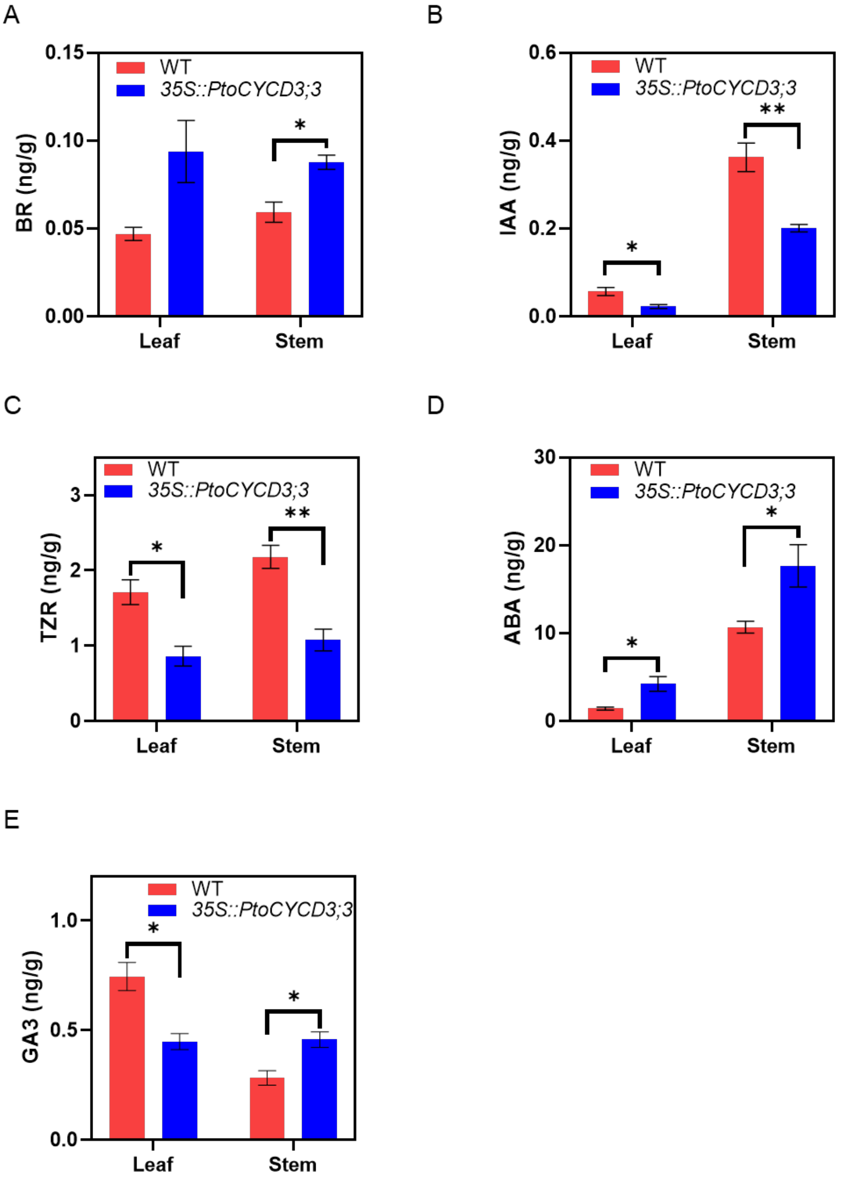

Supplementary Figure S8. Hormone content of wild-type and *35S::PtoCYCD3;3* plants. (A) BR content of leaf and stem; (B) IAA content of leaf and stem; (C) TZR content of leaf and stem;(D) ABA content of leaf and stem; (E) GA3 content of leaf and stem.
