# Supplementary material for: Overexpression of PtoCYCD3;3 Promotes Growth and Causes Leaf Wrinkle and Branch Appearance in Populus
Source: Int J Mol Sci. 2021 Jan 28;22(3):1288. doi: 10.3390/ijms22031288 (PMC7866192; doi:10.3390/ijms22031288)
Supplement: Supplementary file 1 [file ijms-22-01288-s001.zip › Supplementary Figure S9.pdf]

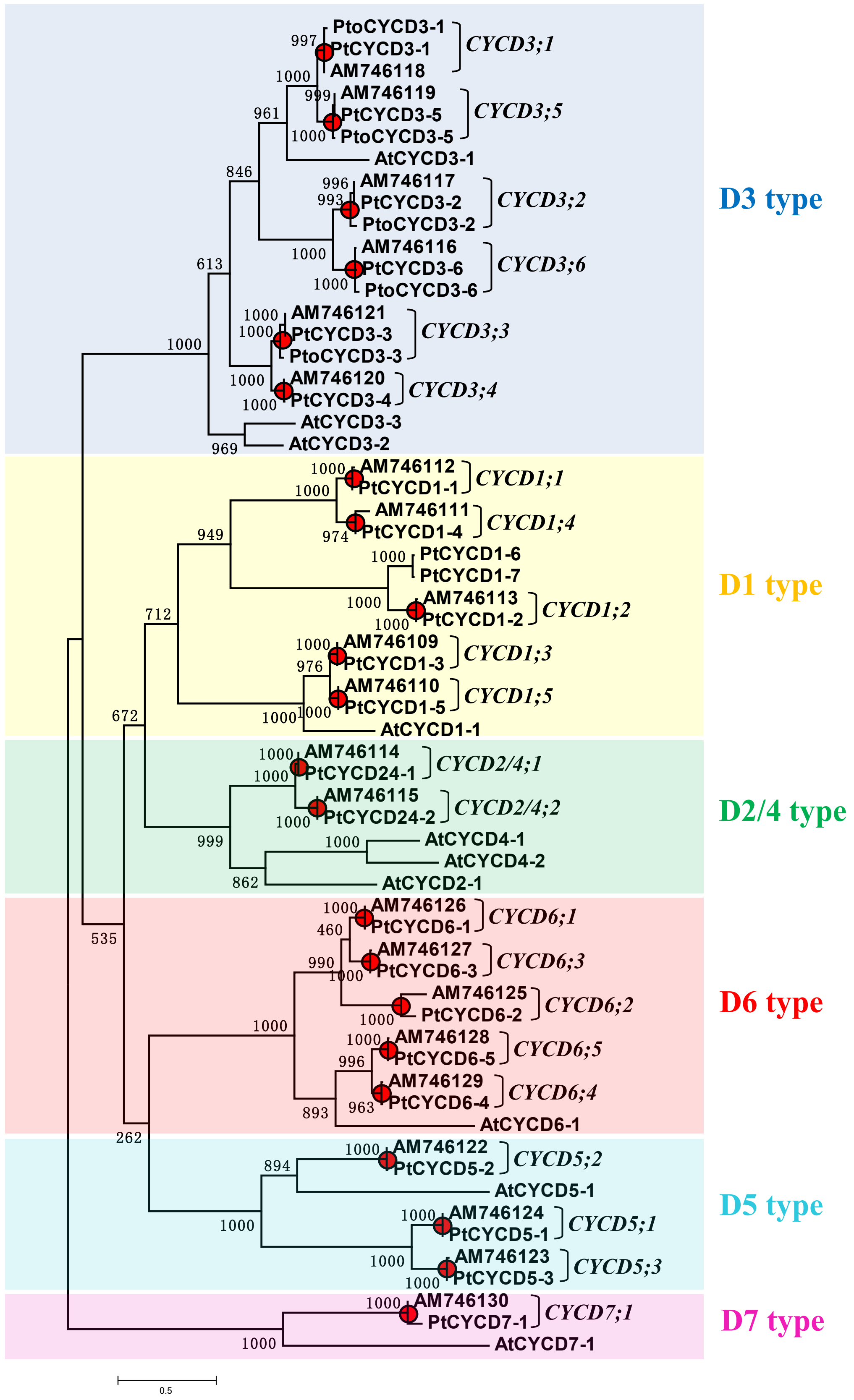

Supplementary Figure S9. Phylogenetic tree of *CYCD* members from *PtCYCD* genes both we identified in *P. trichocarpa* and studied in Menges et al., 2007, *PtoCYCD3* genes we sequenced in *P. tomentosa*, and *AtCYCD* genes in *Arabidopsis*. The orthologous relationships of *CYCD* genes in *Populus* species were marked with red circle and labeled (Detailed gene information please see Supplementary Table S3) . Phylogenetic trees were constructed using maximum-likelihood (ML) method in PHYML software with the Whelan and Goldman (WAG) amino acid substitution model. The proportion of invariable sites (I) and gamma distribution (G) parameter was estimated, and the bootstrap replicates were set to 1000.
