# Supplementary figures and images for: Overexpression of PtoCYCD3;3 Promotes Growth and Causes Leaf Wrinkle and Branch Appearance in Populus
Source: Int J Mol Sci. 2021 Jan 28;22(3):1288. doi: 10.3390/ijms22031288 (PMC7866192; doi:10.3390/ijms22031288)

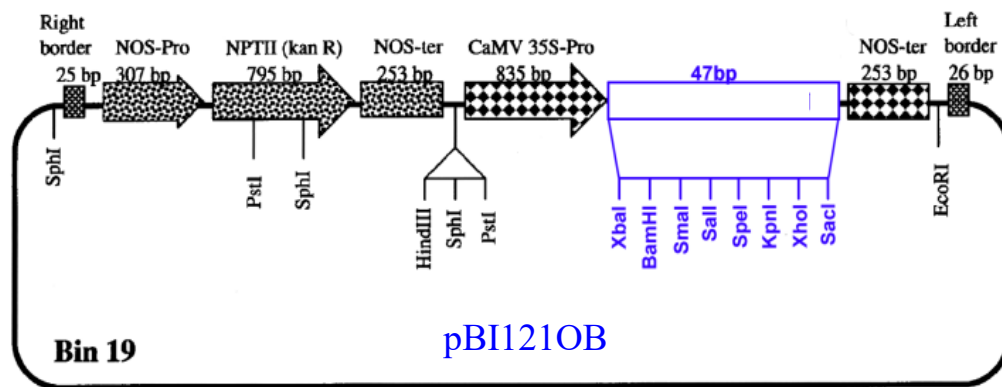

**Supplementary Figure S10.** The pBI121OB vector.

Supplement: Supplementary file 1 [file ijms-22-01288-s001.zip › Supplementary Figure S10.pdf]

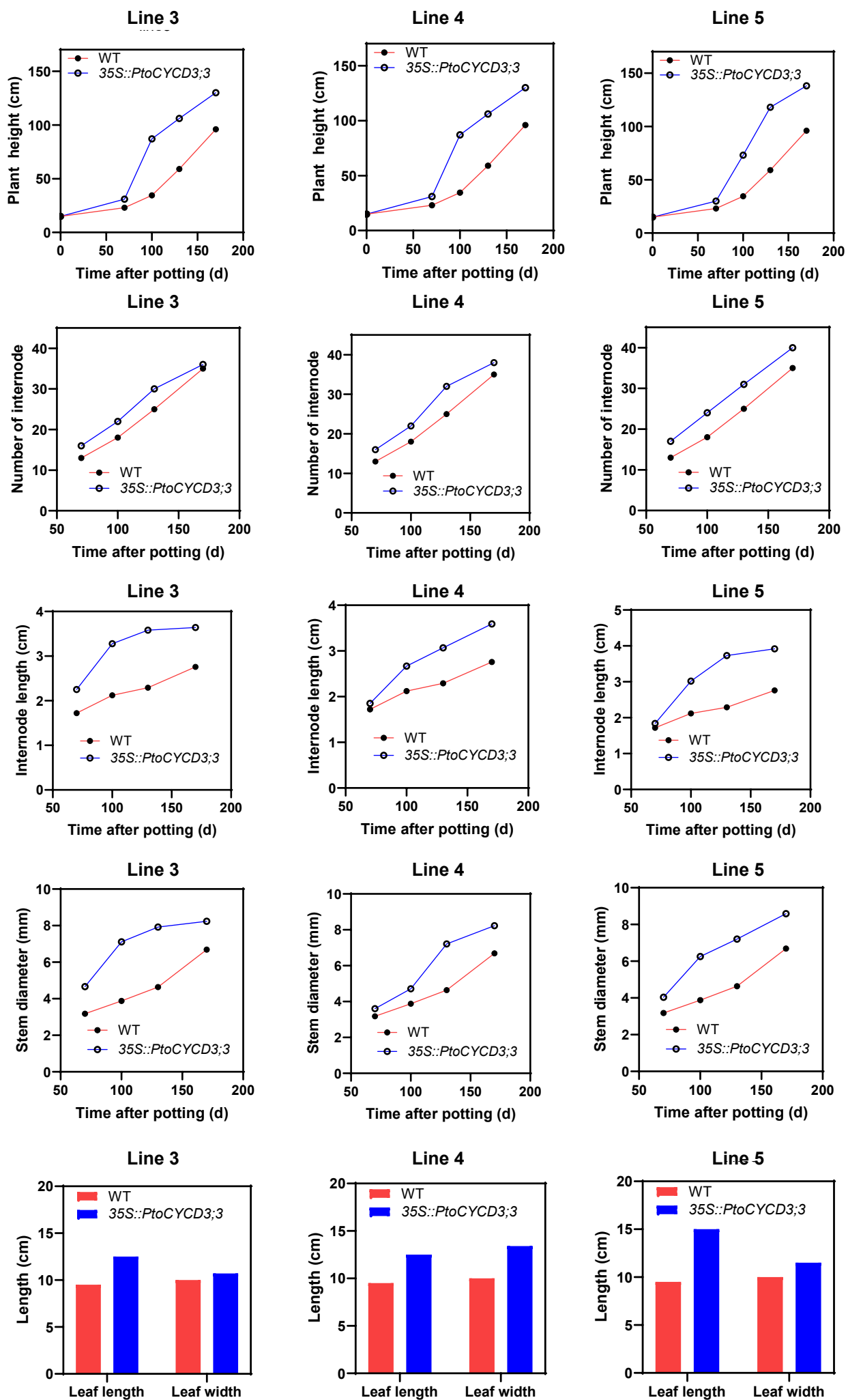

Supplementary Figure S3. Growth parameters of three overexpression lines (Line3, Line4, Line5)

Supplement: Supplementary file 1 [file ijms-22-01288-s001.zip › Supplementary Figure S3.pdf]

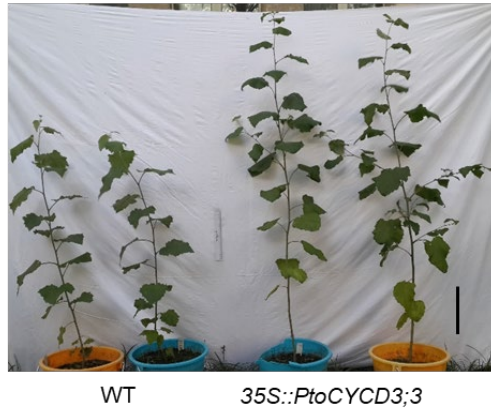

**Supplementary Figure S4.** Phenotypes of wild-type and *35S::PtoCYCD3;3* plants. Bar: 20cm

Supplement: Supplementary file 1 [file ijms-22-01288-s001.zip › Supplementary Figure S4.pdf]
